# The Influence of Pandemic-Related Worries During Pregnancy on Child Development at 12 Months

**DOI:** 10.21203/rs.3.rs-2682358/v1

**Published:** 2023-03-22

**Authors:** Lauren K. White, Megan M. Himes, Rebecca Waller, Wanjikũ F.M. Njoroge, Barbara H. Chaiyachati, Ran Barzilay, Sara L. Kornfield, Heather H. Burris, Jakob Seidlitz, Julia Parish-Morris, Rebecca G. Brady, Emily D. Gerstein, Nina Laney, Raquel E. Gur, Andrea Duncan

**Affiliations:** Children’s Hospital of Philadelphia; Children’s Hospital of Philadelphia; University of Pennsylvania; Children’s Hospital of Philadelphia; Children’s Hospital of Philadelphia; Children’s Hospital of Philadelphia; University of Pennsylvania; Children’s Hospital of Philadelphia; University of Pennsylvania; Children’s Hospital of Philadelphia; Washington University School of Medicine; University of Missouri–St. Louis; Children’s Hospital of Philadelphia; University of Pennsylvania; Children’s Hospital of Philadelphia

**Keywords:** Child development, emotion regulation, pandemic, parent-child interactions, parenting

## Abstract

The COVID-19 pandemic has been linked to increased risk for perinatal anxiety and depression among parents, as well as negative consequences for child development. Less is known about how worries arising from the pandemic during pregnancy are related to later child development, nor if resilience factors buffer negative consequences. The current study addresses this question in a prospective longitudinal design. Data was collected from a sub-study (*n* = 184) of a longitudinal study of pregnant individuals (total *n* = 1,173). During pregnancy (April 17-July 8, 2020) and the early postpartum period (August 11, 2020-March 2, 2021), participants completed online surveys. At 12 months postpartum (June 17, 2021-March 23, 2022), participants completed online surveys and a virtual laboratory visit, which included parent-child interaction tasks. We found more pregnancy-specific pandemic worries were prospectively related to lower levels of child socioemotional development based on parent report (B=−1.13, SE = .43, *p* = .007) and observer ratings (B=−0.13, SE = .07, *p* = .045), but not to parent-reported general developmental milestones. Parental emotion regulation in the early postpartum period moderated the association between pregnancy-specific pandemic worries and child socioemotional development such that pregnancy-specific pandemic worries did not related to worse child socioemotional development among parents with high (B=−.02, SE = .10, t=−.14, p = .89) levels of emotion regulation. Findings suggest the negative consequences of parental worry and distress during pregnancy on the early socioemotional development of children in the context of the COVID-19 pandemic. Results highlight that parental emotion regulation may represent a target for intervention to promote parental resilience and support optimized child development.

## Introduction

Beginning in early 2020, the coronavirus-19 (COVID-19) pandemic upended lives worldwide with major impacts on mortality and morbidity (Stokes, 2020). The pandemic was particularly challenging for pregnant individuals who were considered high risk for more severe symptoms, complications, and mortality from contracting COVID-19 (CDC, 2022; Chmielewska et al., 2021). The inconsistent and continually changing prenatal, labor, and childbirth guidelines also contributed to the distress. Arising concerns included attending prenatal appointments alone, vaccine safety, guests in the delivery room, and testing positive for COVID-19 at admission and subsequently being separated from their newborn after delivery (Kotlar et al., 2021). A growing body of work documents the vast negative consequences of the pandemic on individuals during pregnancy, childbirth, and early postpartum, which are already periods of major stress, change, and uncertainty (Berthelot et al., 2020; Davenport et al., 2020; Gur et al., 2020; Kornfield et al., 2021; Saccone et al., 2020; Wu et al., 2020; Yan et al., 2020). In addition to charting the experiences of perinatal individuals during this universally stressful period, studies are also needed that examine how those experiences may have affected child development, with implications that might extend beyond the current pandemic. Such findings could inform our ability to improve parental and child well-being in the context of social stressors more broadly.

Exposure to extreme or chronic environmental stress early in life, including in utero, has lifelong consequences for child development (Lautarescu et al., 2020; Robinson et al., 2017; Sandman et al., 2012). Prior studies that have investigated the impact of pandemics or natural disasters (e.g., 1918 Influenza Pandemic, Hurricane Katrina, Quebec Ice Storm) have demonstrated transgenerational effects on offspring, including poorer cognitive development, lower educational attainment, and increased risk for adverse mental health outcomes (Masten & Osofsky, 2010; Schoenmakers et al., 2022; K. Squires et al., 2022). Since the onset of the COVID-19 pandemic, studies have established decreases in childhood primary care visits (Macy et al., 2020), more adverse childhood experiences, (Calvano et al., 2022; Chanchlani et al., 2020), and negative child development outcomes (Deoni et al., 2021; Duguay et al., 2022; Shakiba et al., 2022; Shuffrey et al., 2022; Waller et al., 2021). Based on neurodevelopmental scores on the Ages & Stages Questionnaire (ASQ-3), 12-month-old children born during the pandemic showed significantly lower communication, gross motor, fine motor, and personal-social scores when compared to pre-pandemic 12-month-old children (Giesbrecht et al., 2022).

However, there remains a paucity of work that has examined whether pandemic-related stress during pregnancy is prospectively related to later child development disruptions. More broadly, studies conducted prior to the pandemic have established links between perinatal distress and poorer child outcomes including worse cognitive performance (Glover, 2015), psychiatric disorders (Bale et al., 2010; Van den Bergh et al., 2020), and more socioemotional (Porter et al., 2019) and behavioral (Kingston & Tough, 2014) problems. Pregnancy-related anxiety (i.e., specific anxiety about the emotions, thoughts, and physical symptoms experienced by pregnant individuals; Bayrampour et al., 2016), has also been uniquely linked to negative developmental outcomes in offspring, including developmental delays (Shao et al., 2021) and risk for emotional and behavioral problems (Wang et al., 2021). Thus, heightened prenatal pandemic-related anxiety may also signal risk for poorer developmental outcomes in children, although studies have yet to examine such associations within a prospective framework.

Notably, there are likely key resilience factors that could buffer any deleterious impacts of pandemic stress on child outcomes, including emotion regulation (i.e., the ability to effectively modify the experience and expression of emotions; Gross, 1998; Thompson, 1994; Webb et al., 2012). Higher levels of parental emotion regulation have been linked to fewer symptoms of postpartum depression (Kornfield et al., 2021) and to improved child outcomes, including fewer externalizing problems (Byrd et al., 2021) and better emotion regulation (Morelen et al., 2016; Tan & Smith, 2019). However, studies have yet to test whether parental emotion regulation can buffer against the negative effects of prenatal pandemic-related worries on child development.

The current study addresses these knowledge gaps using data from a prospective longitudinal study that began in pregnancy during a statewide stay-at-home order in response to the first wave of the COVID-19 pandemic, with survey data collected during pregnancy, early postpartum, and 12 months postpartum, as well as observational data collected during parent-child interaction tasks assessed virtually at 12 months postpartum. Our first aim was to test whether pandemic-related worries were associated with child socioemotional and general development at age 12 months. To rule out informant method effects, models controlled for maternal mental health in pregnancy (i.e., time of exposure) and 12 months postpartum (i.e., time of outcome). We hypothesized that more pandemic worries would be related to worse child outcomes across methods (i.e., parent report and observational data). Second, we tested whether emotion regulation in the early postpartum period served as a protective factor against the effects of pandemic worries on child development. We hypothesized that emotion regulation would buffer the impact of pandemic worries, such that at high levels of emotion regulation in the early postpartum period, there would be no association between pandemic-related worries and later child outcomes. Finally, in the context of a large literature establishing significantly greater mortality, morbidity, unemployment, and burden arising from the pandemic among Black communities in the US (Gur et al., 2020; Selden & Berdahl, 2020; Tai et al., 2021; US Census Bureau COVID-19 Site, 2022), we ran exploratory post hoc analyses to test whether associations between pandemic worries and child development differed for participants who were Black or Non-Latinx White.

## Methods

### Design and Procedures

Data were from *n* = 184 parent-infant dyads, who were recruited as a sub-study of a larger longitudinal perinatal cohort (see Gur et al., 2020; Kornfield et al., 2021 for information on the full cohort). For the larger cohort, an electronic health record (EHR) search identified pregnant individuals ≥ 18 years receiving prenatal care through Penn Medicine between April 17, 2020, and May 1, 2020. Individuals were invited via email to participate in a REDCap survey at the height of the first lockdown after the World Health Organization (WHO) declared COVD-19 to be a global pandemic (time 1; April 17-July 8, 2020). A postpartum survey was conducted through another REDCap survey at 10–15 weeks postpartum (time 2; August 11, 2020-March 2, 2021). Finally, at 12 months postpartum (June 17, 2021-March 23, 2022; time 3), we began a new sub-study called the Prenatal to Preschool (P2P) study, which recruited a subsample of the original cohort, focusing on Black and Non-Latinx White individuals (race and ethnicity collected through EHR). Data were obtained using two methods: a REDCap survey and a virtual laboratory visit (see Table 1 for constructs measured across time points).

As part of the online visit at 12 months, parents completed an adapted version of the semi-structured Three Bags procedure (Brady-Smith et al., 1999; NICHD Early Child Care Research Network, 1997; Whalen & Gilbert, 2017). Materials for the virtual laboratory visit (i.e., a wordless storybook; two cups and a ball; a toy rattle; and a snack) were shipped to participants. The contents were chosen based on prior literature on in-person studies, with a focus on materials that could be readily mailed and that would stimulate both directive parenting behaviors and child engagement. Ethical Considerations: All study procedures were approved through IRBs at the University of Pennsylvania and Children’s Hospital of Philadelphia. Participants provided consent prior to completing each assessment.

### Participants

There were 1,173 individuals who completed the survey at time 1 (Gestational age [GA], *M* = 24.6 weeks, *SD* = 8.4; see **Table S1**). At time 2, survey data was available for 833 individuals (76% retention; *M* = 11.8 weeks postpartum, *SD* = 4.2). At time 3, the sub-study recruited 219 parent-child dyads (*M*_age_=13.6 months, *SD*_age_=1.5) for a virtual laboratory visit and survey (4 participants only completed the surveys). Participants who completed the child development questionnaire measures outside the recommended window for interpreting scores (range 12–14 months; *n* = 20) and participants with GA < 37 weeks (*n* = 13) were excluded from analyses. Excluded participants showed higher rates of prenatal mental health symptoms and COVID-19 worries compared to the final included sample (*n* = 184), likely driven by pregnancy complications related to preterm birth (see **Table S2**).

### Measures

#### Self-reported pandemic worries (time 1).

During pregnancy, participants reported their level of worry for six general worries about the COVID-19 pandemic (e.g., “dying from COVID-19” or “family members getting COVID-19”; α = .84) and four pregnancy-specific worries about COVID-19 (e.g., “having access to food, meds, and baby care items during the COVID-19 pandemic” and “receiving good prenatal care during the COVID-19 pandemic”; α = .82) (see **Table S3** for a full list of items) (Gur et al., 2020; Kornfield et al., 2021; Njoroge et al., 2021).

#### Self-reported mental health (time 1 and 3).

The Generalized Anxiety Disorder 7 (GAD-7; Spitzer et al., 2006) assessed anxiety symptoms. For depression symptoms, participants completed the Patient Health Questionnaire 2 (PHQ-2; Carey et al., 2016) at time 1 and the Edinburgh Postnatal Depression Scale at time 3 to assess postpartum depression (EPDS; Cox & Holden, 2003). The self-harm EPDS item was excluded given difficulties associated with monitoring self-harm endorsements virtually (Kornfield et al., 2021).

#### Self-reported emotion regulation (time 2).

Emotion regulation was assessed using a 5-item scale (Barzilay et al., 2020; Gur et al., 2020; Kornfield et al., 2021; White et al., 2022), which was part of the Brief Risk and Resilience Battery (Moore et al., 2020). This scale was adapted from the Difficulties in Emotion Regulation Scale (Gratz & Roemer, 2004). Participants completed items that assessed their ability to regulate emotions (e.g., “When I’m upset, I have difficulty controlling my behaviors” and “When I’m upset, I have difficulty concentrating”; α = .84).

#### Parent-reported general developmental milestones (time 3).

The Survey of Well-being of Young Children (SWYC) Milestones (12-month form) was used to assess general developmental milestones (Sheldrick & Perrin, 2013). Response options were not yet, somewhat, and very much, which are used to compute a total score, with higher scores indicating more advanced general development (α = .73).

#### Parent-reported socioemotional development (time 3).

The Ages and Stages Questionnaire: Social-Emotional, Second Edition (ASQ:SE-2; J. Squires et al., 2015) was used to assess socioemotional development. Response options are: often or always, sometimes, and rarely or never. According to the scoring manual, values of 0, 5, or 10 are assigned to the items and calculated for a total score (α = .74). We recoded the scoring such that higher scores indicated better socioemotional development. To reduce participant burden, we removed the follow-up items asking respondents to “check if this is a concern” as the scale was not being used for clinical purposes.

#### Observed socioemotional development (time 3).

A team of trained researchers coded the interactions using an adapted version of the Three Bags procedure. Socioemotional development was derived from ratings of child engagement during the storybook and free play tasks, which both elicited frequent parent-child interaction in a semi-unstructured context. The child engagement construct assesses level of interaction and positive communication with the parent, as evidenced through eye contact, smiling, positively responding to play initiations, or engaging in play. Training for coding involved an introduction to the system and manual followed by meetings to discuss and practice coding training videos. Trainees were required to code 10 or more videos to establish inter-rater reliability before coding independently. Weekly meetings were held by the coding team to maintain fidelity with the manual. Coders watched each task three times before rating the constructs on a scale from 1 (very low) to 7 (very high), with higher scores indexing a greater degree of social engagement.

Videos were available for *n* = 132 based on the free play task and *n* = 138 for the story book task, with *n* = 113 participants having data available for both tasks and *n* = 27 participants having no video available data. The most common reasons for exclusion were child or parent face/body out of view. Participants with no available video data did not differ significantly from participants with video data from one or both tasks on key study variables, including pregnancy-specific pandemic worries, general pandemic worries, anxiety symptoms, or depression symptoms in pregnancy (all *ps* > .31). Inter-rater reliability was calculated on a random 20% of videotapes stratified across coders using the intra-class correlation coefficient (ICC) with absolute agreement. ICC is considered a conservative estimate of reliability because it corrects for chance agreement and takes into consideration both rank order and absolute distance between two scores (Shrout & Fleiss, 1979). Inter-rater reliability was high for social engagement for both the free play (ICC = .84) and storybook (ICC = .80) tasks. Scores were moderately correlated (*r* = .39, *p* < .001) and were combined into a single observed socioemotional development measure to reflect socioemotional behaviors over multiple contexts.

#### Demographic covariates.

Covariates included child age in months, child sex, parental age, parity, and EHR race (Table 2). Socioeconomic disadvantage was also included as a covariate, which was a composite scale derived from census-based geocoding of neighborhood-level variables (e.g. percent in poverty, percent married, and median family income) based on parent-reported zip code (Moore et al., 2016). Socioeconomic disadvantage was included given documented links with adverse child development (Betancourt et al., 2016; Gottfried et al., 2003; Luby et al., 2022; Murtha et al., 2022). We re-ran models using an alternative measure of socioeconomic disadvantage, parent-reported income from time 2 (“During the last year, what is your household income before taxes, from all sources?”).

### Analytic Strategy

Study aims were tested within a multivariate framework in Mplus vs. 8.0 (Muthén & Muthén, 1998–2017). All participants with complete or partial data, including video data (*n* = 184), were included in analyses using full-information maximum likelihood estimation with robust standard errors or maximum likelihood estimation (Enders & Bandalos, 2001). To address our first aim, we tested a path model that specified the three child development outcomes the 12-month visit – parent-reported and observed socioemotional development and parent-reported developmental milestones – as correlated dependent variables. We entered pregnancy-specific and general pandemic worries as independent variables in the model and controlled for child age, child sex, maternal age, parity, race, and socioeconomic disadvantage, as well as the covariance of predictors. To establish specificity of the findings to pandemic worries and rule out informant method effects or the effect of psychiatric symptoms on reporting biases, we re-ran the model controlling for anxiety and depression at time 1 (i.e., at time of risk exposure) and controlling for anxiety and depression at time 3 (i.e., time of outcome assessment). We also re-ran the model to establish the robustness of findings contingent on how socioeconomic disadvantage was specified (neighborhood-based socioeconomic index or parent-reported annual income). To address our second aim to investigate emotion regulation at time 2 as a potential resilience factor, we first entered the main effect of emotion regulation within path models. Next, we created and entered mean-centered and product terms between emotion regulation, general pandemic worries, and pregnancy-specific pandemic worries. We added these terms to the model predicting the correlated dependent variables of parent-reported and observed socioemotional development and parent-reported developmental milestones. Significant interactions were probed following recommended guidelines using an online tool (Preacher et al., 2006) and plotted in R (RStudio Team, 2019).

## Results

### Descriptive Statistics

Table 2 presents descriptive statistics for study variables from the P2P sub-study assessed in the current study. Participants were from the following racial groups: Non-Latinx White (*n* = 96, 52.2%) and Black (*n* = 88, 47.8%), with *n* = 3 (1.6%) individuals identified as Black and Latinx per EHR. At time 1, 7.6% individuals were in the first trimester, 51.6% in the second trimester, and 40.8% in the third trimester. There were low-to-moderate significant bivariate correlations (Table 3) between parent-reported measures within and across constructs both cross-sectionally and over time, justifying our approach of using path models capable of accounting for covariances of independent and dependent variables.

### Aim 1: Pandemic-related Worries During Pregnancy And Child Development At 12 Months

In a path model with all three 12 month developmental outcomes specified as correlated dependent variables and controlling for abovementioned covariates, higher pregnancy-specific pandemic worries in pregnancy were related to lower socioemotional development in children based on both parent-report (B=−1.13, SE = .43, *β*=−.21, *p* = .007) and observer ratings (B=−.13, SE = .07, *β*=−.21, *p* = .045), but not to parent-reported general developmental milestones (B=−.06, SE = .07, *β*=−.08, *p* = .34). Although general pandemic worries were correlated with lower parent-reported socioemotional development in bivariate models (Table 3), the association was not significant when pregnancy-specific pandemic worries were included as a predictor within models. General pandemic worries were unrelated to observed socioemotional development and general developmental milestones (Table 4). In terms of covariates, lower parent-reported socioemotional development was reported by participants experiencing more socioeconomic disadvantage (*B*=−4.89, *SE* = 1.62, *β* = .23, *p* = .001) and who were identified as Black in their EHR (*B*=−6.15, *SE* = 3.14, *β* = .15, *p* = .04). Older children were reported to have achieved more general developmental milestones (*B* = .31, *SE* = .08, *β* = .29, *p* < .001).

To account for informant method effects, including the possibility that symptoms of depression and anxiety could influence parent reports of child development or interactions with children during the virtual visits, we showed that findings linking pregnancy-specific worries to both parent-reported and observed child socioemotional development were unchanged after controlling for anxiety and depression in pregnancy (**Tables S4** and **S5**) and concurrent anxiety and depression at 12 months postpartum (**Tables S6** and **S7**). Depression (*B*=−.94, *SE* = .33, *β*=−.21, *p* < .001) and anxiety (*B*=−.61, *SE* = .30, *β*=−.13, *p* = .04) assessed concurrently at 12 months were also related to lower parent-reported socioemotional development. The pattern of findings was similar when we used individual reports of income (**Table S8**). There were no significant interaction terms between general or pregnancy-specific pandemic worry scores and parent race in relation to any developmental outcome (**Table S9**); the associations between pandemic worries and child developmental outcomes did not vary on the basis of race.

### Aim 2: Emotion Regulation In The Postpartum Period As A Resilience Factor

**Figure 1:**
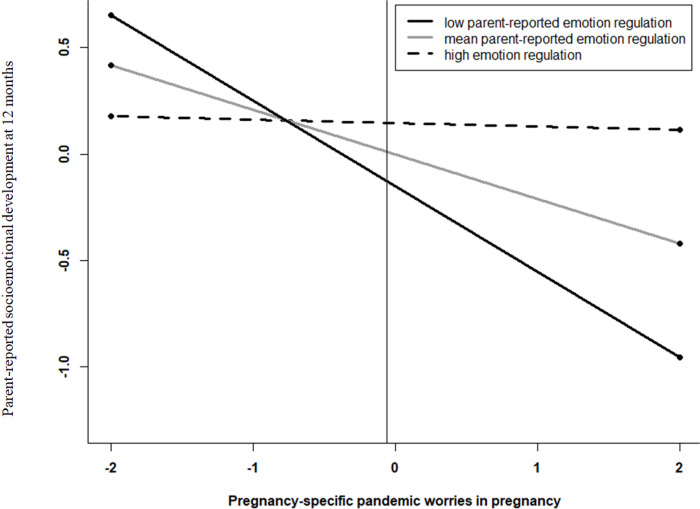
High levels of emotion regulation in the early postpartum period buffer against the effect of pandemic worries on parent-reported socioemotional development

We examined whether parent emotion regulation in the postpartum period, as a component of resilience, buffered the risk pathway between pandemic-related concerns during pregnancy and poor child developmental outcomes, controlling for covariates as before. First, there were main effects such that greater emotion regulation in postpartum was associated with better parent-reported socioemotional development (*B* = .90, *SE* = .36, *β* = .19, *p* = .01) and general developmental milestones (B = .13, SE = .06, β = .17, *p* = .02), though no association was found with observed child socioemotional development (Table 5a). Second, there was a significant interaction between pregnancy-specific worries and maternal emotion regulation in the postpartum period specifically in relation to parent-reported socioemotional development (*B* = .22, *SE* = .09, *β* = .19, *p* = .02) (Table 5b). Probing this interaction revealed that more pregnancy-specific pandemic worries were only related to lower socioemotional development at age 12 months when parents reported low (*B*=−.40, *SE* = .12, *t*=−3.60, *p* < .001) or mean (*B*=−.21, *SE* = .08, *t*=−2.75, *p* = .01) levels of emotion regulation in the postpartum period. At high levels of emotion regulation, the link was buffered (i.e., protective effect; *B*=−.02, *SE* = .10, *t*=−.14, *p* = .89) (see Fig. 1).

## Discussion

The current study examined how an extreme stressor experienced during pregnancy was related to children’s development at 12 months of age. Our study occurred within the context of the COVID-19 pandemic and highlights the many challenges experienced by pregnant individuals (Abrams et al., 2022; Giesbrecht et al., 2022; Yan et al., 2020). We compared associations between general pandemic worries (e.g., dying from COVID-19) and pregnancy-specific pandemic worries (e.g., getting exposed to the virus that causes COVID-19 during prenatal care visits) at the beginning of the pandemic and later child development, leveraging both report and observed measures within a prospective longitudinal design. First, among parents who reported more pregnancy-specific worries during pregnancy, children had poorer socioemotional development based on both parent reports and observed ratings. Second, parental emotion regulation during the postpartum period served as a significant protective factor against the risk posed by prenatal pandemic-related worries. Our findings provide a critical quantitative narrative about the experiences of pregnant individuals during this time of significant global stress and uncertainty, while offering important insights into protective factors that could be targeted in interventions more broadly to minimize links between stress exposure in pregnancy and child development.

More worries during pregnancy about how the COVID-19 pandemic would affect prenatal care, childbirth experience, and aspects of caring for their newborn, was linked to significantly lower levels of both parent-reported and observed socioemotional development in children. Our results support prior findings from a similar-aged sample suggesting disruptions to normative development in children born during the pandemic (Giesbrecht et al., 2022) and extend the results of other prior studies focused on younger infants, including a reported link between general prenatal distress during the pandemic and poorer socioemotional development in 2-month-old children (Duguay et al., 2022). Interestingly, our findings were specific to pregnancy-related pandemic worries. That is, although there had been a bivariate association between general pandemic worries and child developmental outcomes, the association was rendered non-significant when pregnancy-specific worries were entered into the model. Prior studies conducted in the larger cohort from which our sub-study was drawn have reported that pregnancy-specific worries are uniquely linked to screening positive for both prenatal and postpartum depression (Gur et al., 2020; Kornfield et al., 2021). Taken alongside the current results, our measure of pregnancy-specific pandemic worries may therefore suggest an index of the broader construct of pregnancy-related anxiety, which has robust and independent links to poorer maternal-infant bonding and child development (Bayrampour et al., 2016; Hadfield et al., 2022), and has been established as a separate construct from general anxiety. Prior studies have linked pregnancy-related pandemic worries to measures of pregnancy-related anxiety (Lebel et al., 2020; Moyer et al., 2020). Thus, it may be important for clinicians to evaluate pregnancy-specific measures of distress over and above measures of general anxiety during the perinatal period, especially in the context of stress exposure (i.e., pandemic in this case).

Notably, scores on the measure of pregnancy-specific pandemic worries were not associated with parent-reported general developmental milestones. One explanation for why pregnancy-specific pandemic worries were only linked to child socioemotional development is that the pregnancy-specific worries we identified could have subsequently evolved to parenting-related worries after birth during the transition to parenthood or the addition of a new child to the family. Increased stress or worry around parenting could have then inadvertently stymied positive parenting practices (e.g., lower parental sensitivity, nurturance, or involvement), which have specifically been shown to influence children’s socioemotional development (Behrendt et al., 2019). Another explanation is that the pandemic negatively impacted the social milieu of parents, conferring greater isolation and loneliness (Li et al., 2021), or fewer social interactions for the developing infant to have observed, modeled, or imitated (Bandura, 1977; Woszidlo & Kunkel, 2017). Although outside the scope of the current study to test, pregnancy-related worry may also have altered the neural or biological architecture underlying social and emotional functioning in the developing fetus (Madigan et al., 2018; Miguel et al., 2019; O’Donnell & Meaney, 2017). Finally, the lack of a significant association between pandemic worries and general developmental milestones may be a consequence of being underpowered with our sample size or the limited age range of children, which restricted individual differences in our measure. Further research in larger samples assessed across wider age ranges is needed to further explore the specificity of pregnancy-related pandemic stress on child development.

Importantly, we found that higher levels of emotion regulation in the early postpartum period related to positive socioemotional development and buffered against the negative effect of pregnancy-specific pandemic worries. This finding is consistent with a growing area of research demonstrating that greater parental emotion regulation is associated with benefits for child emotional development (Hajal & Paley, 2020; Morris et al., 2007). There are several different pathways through which parental emotion regulation could foster better socioemotional development in children. For example, greater emotion regulation skills could directly or indirectly support more positive parenting practices (Crandall et al., 2015; Morris et al., 2013), including parental sensitivity, consistency, and involvement, which have been linked to better socioemotional skill development in children (Behrendt et al., 2019; Cprek et al., 2015). Alternatively, there may be intergenerational transmission of emotion regulation from parents to children (Tan & Smith, 2019), which likely fosters better socioemotional processing and competencies in the child. These pathways likely exert a protective force in the presence of high prenatal stress (e.g., pregnancy-related pandemic worries).

There are a few limitations to the current study. First, we relied for the most part on parent report measures, which could have introduced shared method variance (i.e., between reports of pandemic-related worries and child development) or rendered our analyses subject to informant biases resulting from psychiatric comorbidities. We addressed these issues by including an observational measure of child socioemotional development (i.e., observer ratings of engagement) and by controlling for symptoms of depression and anxiety at the time of pandemic worries being reported and child outcomes being reported. Nevertheless, future studies that leverage clinical assessments and reports from alternate caregivers are needed, especially since our measures of parent-reported and observed socioemotional development were not significantly correlated. Second, although we focused on a sample with approximately equal numbers of Non-Latinx White and Black participants, our sample is not representative of the wider population and findings may not generalize to other ethnic and racial groups, including those also negatively impacted by the pandemic (e.g., Latinx, American Indian or Alaska Native, and Native Hawaiian or Other Pacific Islander; Kaiser Family Foundation, 2022). Moreover, we collected race and ethnicity data via EHR, which can differ from self-reported race. Third, we excluded participants with preterm birth since it is a major contributor to neurodevelopment (Adams-Chapman & Stoll, 2006). Thus, our findings may only generalize to full-term cohorts. Fourth, we were unable to examine if the current pattern of results differed as a function of exposure timing (trimester at which worries were reported) as there were few participants that completed the Time 1 assessment in the first trimester. Finally, we did not incorporate a standard measure of pregnancy-related anxiety (Bayrampour et al., 2016; Hadfield et al., 2022), so it is unclear if the significant effects of our pandemic pregnancy-specific worries measure were truly due to worries surrounding being pregnant during COVID-19 or general pregnancy-related anxiety.

In conclusion, our findings add to a growing body of literature that has both specific and general takeaways for perinatal mental health and early childhood development. In terms of specificity, we show that the lived experience of the COVID-19 pandemic may have put children at risk for less optimal developmental outcomes, particularly in the context of a parent experiencing a high degree of worry about being pregnant and caring for a newborn during the pandemic. Further work is needed to establish whether this generation of children – *those born during a pandemic* – will need additional intervention efforts to ensure they do not remain behind in socioemotional or general development. In terms of generalities, the findings have implications for how we might address and minimize the impacts of stress exposure more broadly during pregnancy (e.g., future pandemic, environmental disasters, war) on parents and children. In particular, we need rigorous testing of prevention and intervention efforts that focus on building maternal resilience through better emotion regulation (e.g., Havighurst et al., 2013; Nunnery et al., 2021; Porzig-Drummond et al., 2014), which could bring benefit to both parental mental health and child development.

## Figures and Tables

**Table 1:** Summary of Measures across Time Points

	Demographic covariates	Pandemic worries	Mental health	Resilience	Child development outcomes
**Time 1** (*pregnancy*; self-report survey and collected from electronic health records)	Maternal age Gestational age Socioeconomic disadvantage Parity Race	General pandemic worries Pregnancy-specific pandemic worries	Anxiety Depression		
**Time 2** (*10–15 weeks postpartum*; self-report survey)				Emotion regulation	
**Time 3** (*12 months postpartum*; parent-report survey and virtual laboratory visit)	Child age Child sex		Anxiety Postpartum depression		General developmental milestones Socioemotional development (parent-report) Socioemotional development (observed)

**Table 2:** Sample Descriptive Statistics

	Range	*M (SD)*
***Pregnancy (time 1)***		
Maternal age (years)	19–46	32.5 (5.2)
Gestational age (weeks)	10–40	25.0 (8.0)
Socioeconomic disadvantage	−2.60–2.08	−.09 (.99)
Postpartum (time 2)		
Postpartum weeks	8–35	12.05 (4.51)
12 months (time 3)		
Child Age (months)	12–14.96	13.20 (.71)
	n	**%**
Pregnancy (time 1)		
**Race**		
Black	88	47.8%
White	96	52.2%
**Parity at Pregnancy Survey**		
Nulliparous	89	48.4%
Postpartum (time 2)		
**Annual income**		
Less than $20,000	25	13.6%
$20,000 to $60,000	41	22.3%
$60,000 to $100,000	22	12.0%
More than $100,000	94	51.1 %
Did not report/missing	2	1.1 %
12 months (time 3)		
**Child sex**		
Female	93	50.5%

The above assessment timings indicate time of variable ascertainment. Socioeconomic disadvantage was a z-score composite scale derived using census-based geocoding neighborhood-based variables (e.g., median family income, residents married, residence with at least a high school education, percent of residence in poverty) using participant addresses. A composite score was created based on the census-based variables (Moore et al., 2016) ranging from − 2.60 to 2.08 with higher values indicating more neighborhood disadvantage. Models included socioeconomic disadvantage as a covariate, but results were similar when we controlled, instead, for annual income reported at the postpartum survey.

**Table 3:** Descriptive Statistics and Bivariate Correlations of Main Study Variables

			*Time 1*				*Time 2*	*Time 3*			
			------------------------------------------------				-------------	-------------------------------------			
	*M*	*SD*	General pandemic worries	Pregnancy-specific pandemic worries	Dep	Anx	Emotion regulation	Dep	Anx	SWYC	ASQ:SE
Pregnancy (time 1) – parent-report											
General pandemic worries	16.30	5.55									
Pregnancy-specific pandemic worries	12.15	3.90	.54***								
Depression	.90	1.36	.26***	.18*							
Anxiety	4.74	4.07	.37***	.26***	.74***						
Postpartum (time 2) – parent-report											
Emotion regulation	18.56	4.47	−.18*	−.14	−.21**	−.18*					
12 months (time 3) – parent-report											
Depression	5.83	4.65	.23***	.24**	.37***	.46***	−.43***				
Anxiety	4.20	4.54	.26***	.30***	.36***	.49***	−.39***	.80***			
General Developmental Milestones (SWYC)	15.66	3.35	−.09	−.09	−.06	−.05	.20*	−.01	.002		
Socioemotional development (ASQ:SE, reverse-scored)	242.20	22.22	−.20**	−.25***	−.15	−.06	.20**	−.25**	−.16*	−.32***	
12 months (time 3) – observed											
Socioemotional development (observer ratings of social engagement)	7.98	2.38	.04	−.10	.10	.03	−.04	−.03	−.04	.18*	.04

Note.

* p < .05

** *p* < .01

*** *p* < .001.

Dep = Depression as measured by PHQ-2 at time 1 and EPDS at time 3; Anx = Anxiety as measured by GAD-7; SWYC = Survey of Well-being of Young Children; ASQ:SE = Ages and Stages Questionnaire: Social-Emotional.

**Table 4:** More pregnancy-specific pandemic worries were prospectively related to lower parent-reported and observed socioemotional development in children at 12 months old, but not general developmental milestones

	Parent-reported socioemotional development				Observed socioemotional development				Parent-reported general developmental milestones			
	B	SE	β	p	β	p	β	p	B	SE	β	p
Child age	.94	.55	.14	.07	−.04	.07	−.05	.59	**.31**	**.08**	**.29**	**<.001**
Child sex	4.92	2.88	.12	.08	−.24	.46	−.05	.60	.23	.47	.03	.63
Socioeconomic disadvantage	**−4.89**	**1.62**	**−.23**	**<.001**	.05	.24	.02	.84	−.22	.29	−.07	.45
Parent age	−.47	.38	−.12	.19	−.04	.06	−.09	.46	−.09	.06	−.14	.10
Parent race	**−6.15**	**3.14**	**−.15**	**.04**	.58	.58	.12	.31	.27	.54	.04	.62
Parity	−1.71	1.08	−.10	.10	.27	.25	.14	.26	.31	.21	.11	.15
General pandemic worries	−.22	.36	−.06	.54	.06	.05	.15	.21	−.03	.06	−.05	.63
Pregnancy-specific pandemic worries	**−1.13**	**.43**	**−.21**	**.01**	**−.13**	**.07**	**−.21**	**.045**	−.07	.07	−.08	.34

*Note.* We examined relationships between general and pregnancy-specific pandemic worries and the three child outcomes within a single path model that modeled the significant correlations between some of the child outcomes (i.e., observed socioemotional development and parent-reported milestones, *r* = .18, *p* = .029; parent-reported milestones and parent-reported socioemotional development, *r* = .31, *p* < .001). Parents who were older had less socioeconomic disadvantage (*r*=−.32, *p* < .001) and greater parity (*r* = .20, *p* < .001). Parents who were Black had greater socioeconomic disadvantage (*r* = .48, *p* < .001), were younger (*r*=−.27, *p* < .001), and had greater parity (*r* = .20, *p* = .002). Higher pregnancy-specific and general pandemic worries were correlated (*r* = .54, *p* < .001). Higher pregnancy-specific pandemic worries were reported among younger (*r*=−.18, *p* = .01) and Black (*r* = .17, *p* = .02) participants.

**Table 5:** Emotion regulation in the early postpartum period is related to socioemotional development in children at 12 months old and buffers against the link between pregnancy-specific pandemic worries and lower socioemotional development

**a.**	***Parent-reported socioemotional development***				***Observed socioemotional development***				***Parent-reported general developmental milestones***			
	*B*	*SE*	*β*	*p*	*B*	*SE*	*β*	*p*	*B*	*SE*	*β*	*p*
Child age	.84	.54	.12	.09	−.04	.07	−.05	.61	**.31**	**.08**	**.28**	**<.001**
Child sex	4.27	2.76	.10	.11	−.19	.45	−.04	.67	.13	.47	.02	.78
Socioeconomic disadvantage	**−5.57**	**1.61**	**−.26**	**<.001**	.08	.25	.03	.75	−.31	.28	−.09	.26
Parent age	−.50	.37	−.13	.16	−.04	.06	−.09	.48	−.10	.06	−.15	.07
Parent race	−5.33	3.06	−.13	.07	.61	.58	.13	.28	.37	.54	.06	.49
Parity	−2.02	1.08	−.12	.05	.28	.24	.14	.25	.27	.20	.10	.18
General pandemic worries *(pregnancy)*	−.11	.34	−.03	.74	.06	.05	.14	.22	−.01	.06	−.01	.89
Pregnancy-specific pandemic worries *(pregnancy)*	**−1.10**	**.42**	**−.21**	**.01**	**−.14**	**.07**	**−.23**	**.03**	−.07	.07	−.08	.35
Emotion regulation (*early postpartum)*	**.90**	**.36**	**.19**	**.01**	−.03	.04	−.05	.53	**.13**	**.06**	**.17**	**.02**
**b.**	***Parent-reported socioemotional development***				***Observed socioemotional development***				***Parent-reported general developmental milestones***			
	*B*	*SE*	*β*	*p*	*B*	*SE*	*β*	*p*	*B*	*SE*	*β*	*p*
Child age	.78	.52	.12	.11	−.04	.07	−.05	.61	**.31**	**.08**	**.28**	**<.001**
Child sex	2.69	2.54	.07	.29	−.22	.48	−.05	.65	−.07	.47	−.01	.89
Socioeconomic disadvantage	**−5.83**	**1.55**	**−.28**	**<.001**	.07	.25	.03	.77	−.33	.28	−.10	.25
Parent age	−.53	.35	−.13	.12	−.04	.06	−.09	.48	−.10	.06	−.16	.07
Parent race	−4.91	3.01	−.12	.09	.67	.58	.14	.24	.43	.54	.06	.42
Parity	−1.25	1.09	−.07	.25	.30	.25	.15	.21	.36	.20	.13	.08
General pandemic worries *(pregnancy)*	−.25	.34	−.07	.46	.06	.05	.13	.28	−.02	.06	−.04	.70
Pregnancy-specific pandemic worries *(pregnancy)*	**−1.11**	**.41**	**−.21**	**.01**	**−.15**	**.06**	**−.24**	**.02**	−.07	.07	−.09	.31
Emotion regulation (*early postpartum)*	**.69**	**.32**	**.15**	**.02**	−.04	.05	−.08	.38	.11	.06	.14	.06
General pandemic worries × Emotion regulation *(interaction)*	.07	.09	.09	.46	.00	.01	.00	.98	.01	.01	.12	.30
Pregnancy-specific worries × Emotion regulation *(interaction)*	**.22**	**.09**	**.19**	**.02**	.01	.02	.09	.42	.02	.02	.08	.46

*Note.* **Part a.** Main effects model showing emotion regulation in the early postpartum period is related to higher parent-reported and observed socioemotional development in children at 12 months old, but not general developmental milestones **Part b.** Model with interaction term, showing emotion regulation interacts with pregnancy-specific pandemic worries in predicting parent-reported socioemotional development

## Data Availability

All data can be made available upon request
